# Misidentification of *Scedosporium boydii* Infection as Aspergillosis in a Patient with Chronic Renal Failure

**DOI:** 10.1155/2020/9727513

**Published:** 2020-06-21

**Authors:** Yuying Jiang, Amira F Gohara, Robert E. Mrak, Kenneth L. Muldrew

**Affiliations:** ^1^Department of Pathology, University of Toledo Medical Center, Toledo, OH 43614, USA; ^2^Department of Pathology and Immunology, Baylor College of Medicine, Houston, TX 77030, USA

## Abstract

Aspergillosis is a commonly diagnosed fungal infection. Histopathologic examination alone can have diagnostic pitfalls due to the overlapping of fungal morphology. We report a case of *Scedosporium boydii* infection initially misdiagnosed as aspergillosis. The patient presented to the hospital with shortness of breath and chest and abdominal pain. Laboratory tests revealed leukocytosis and elevated serum liver enzymes, myoglobin and lipase. He died of hypotensive shock and brain abscesses despite antibiotic treatment. Autopsy revealed invasive fungal infection in the heart, thyroid, and brain with presence of 45-degree angled, branching hyphae. The initial diagnosis of aspergillosis was made; however, further molecular studies identified the organism as *S. boydii*. This report reveals the potential pitfalls of morphologic diagnosis alone; and the necessity of other testing modalities to render an accurate diagnosis which is crucial for appropriate.

## 1. Introduction

Over the last two decades, there have been an increased number of life-threatening systemic fungal infections due to both pathogenic and opportunistic fungi. High-risk populations include immunocompromised patients (AIDS, transplant, malignancy, and immunosuppressive therapy). One of the most frequently encountered filamentous fungal agents is *Aspergillus* species. In recent years, other filamentous fungal pathogens have emerged, such as species of *Fusarium*, *Penicillium*, *Rhizopus*, *Rhizomucor, Scedosporium boydii* (*Pseudallescheria boydii*), and agents of phaeohyphomycosis [1–3]. Although *Aspergillus* remains the most common filamentous mold, the differentiation from other emerging filamentous fungal agents is important due to different treatment regimens. For example, innate resistance or erratic susceptibility to amphotericin B is a characteristic of *S. boydii* [4, 5]. In tissue sections, the characteristic morphologic appearance of the fungal hyphae has been used to help to differentiate *Aspergillus* and *Mucorales*. However, morphology is not always specific. Other diagnostic modalities including fungal culture and fungal ribosomal rRNA gene sequencing are necessary for a definitive diagnosis of the causative fungal agent. Here, we report an autopsy case of systemic invasive fungal infection in a patient with chronic renal failure. Blood and lung cultures submitted at the time of autopsy were negative. Microscopic examination of tissue sections showed the presence of 45-degree angle branching fungal elements. Initial autopsy diagnosis was attributed to systemic aspergillosis. Subsequent broad-range fungal PCR and sequence analysis of DNA extracted from paraffin tissue sections revealed *S. boydii* (*P. boydii)* as the causative agent of the infection. Differentiation of *S. boydii* from *Aspergillus* species is very important for patient management so that the right antifungal agents can be administered.

## 2. Case Report

**Figure 1:**
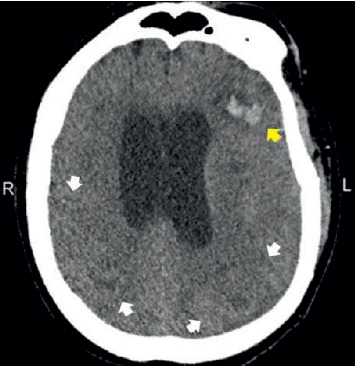
CT scan of the brain showing multiple areas of decreased attenuation in the bilateral occipital lobes and the right parietal lobe (white arrows); subacute intraparenchymal hemorrhage is present in the left frontal lobe (yellow arrow). R, right lateral; L, left lateral.

The patient was a 53-year-old male who had stage V chronic renal disease and was on hemodialysis. He presented to the emergency department with acute respiratory failure, chest pain, and one week history of worsening abdominal pain, nausea, and vomiting. The patient's past medical history included coronary artery disease, stage V chronic kidney disease on hemodialysis, hypertension, diabetes mellitus, warm autoimmune hemolytic anemia, hypercholesterolemia, and gout. His past surgical history included two previous coronary artery stent placements, knee surgery, right upper extremity arteriovenous (AV) fistula, and a recent surgery for a new AV fistula in the left upper extremity one month prior to admission. Following admission, the patient was intubated, and initial laboratory tests were remarkable for leukocytosis, elevated liver enzymes with a negative hepatitis panel, and increased myoglobin, lipase, and creatinine. In addition, the patient exhibited azotemia, metabolic disturbance, and mild coagulopathy (Table 1). Chest X-ray showed cardiomegaly. Despite multidrug antibiotic therapy including vancomycin, Primaxin, doxycycline, and micafungin, the patient's condition continued to worsen, with the white blood cell count increasing from 22,500/mm^3^ to 61,000/mm^3^. The patient's blood pressure dropped precipitously, and he was treated with vasopressive drugs. The patient also had diarrhea; a subsequent colonoscopy was negative. Other laboratory tests were all normal including blood and sputum cultures, serology for CMV, *Brucella* species, *Rickettsia rickettsii*, serum alpha-fetal protein, smooth muscle antibody, mitochondrial antibody, antinuclear antibody, and direct Coombs test. With persistent hypotension, the patient developed atrial fibrillation and had decreased responsiveness. A brain CT scan was performed and showed multiple areas of decreased attenuation with focal mass effect and subacute intraparenchymal hemorrhage in the left frontal lobe (Figure 1). The differential diagnosis of the brain lesions included subacute infarcts and tumor metastasis. The patient died 20 days after admission without a definitive diagnosis of infection.

## 3. Materials and Methods

### 3.1. Histopathologic Study

Numerous specimens from different organ systems were taken at the time of autopsy, fixed in 10% formalin and paraffin embedded. Paraffin sections were cut at 5 *μ*m thickness, stained with hematoxylin eosin (HE), Gomori-Grocott's methenamine silver (GMS), and Gram stains.

### 3.2. Molecular Identification

Paraffin-embedded tissues from the brain, heart, and thyroid lesions with the microscopic presence of fungal elements were studied. Deparaffinized sections were processed using the QIAamp DNA FFPE Tissue Kit (QIAGEN, Hilden, Germany) for DNA extraction. DNA sequencing for identification was performed by analysis of internal transcribed spacer region 2 (ITS2) and the 28S rRNA gene. Polymerase chain reactions (PCR) were performed using the primer pairs 5.8S-F (GTG AAT CAT CGA RTC TTT GAA C) and 28S-1 R (TAT GCT TAA GTT CAG CGG GTA) and D1/2-R (GGT CCG TGT TTC AAG ACG G) as previously described using 30–160 ng of extracted DNA [5]. The presence of correctly sized products was confirmed by agarose gel electrophoresis. Sequencing reactions were performed using purified PCR products and the primers 5.8S-F and D1/2-R. Sequencing was performed with an Applied Biosystems 3500 Genetic Analyzer (Life Technologies). The resulting sequence (GenBank accession number-KJ020683.1) was analyzed phylogenetically using NCBI GenBank Basic Local Alignment Search Tool (BLASTn) to identify the closest database match to the patient's sequence.

## 4. Results

**Figure 2:**
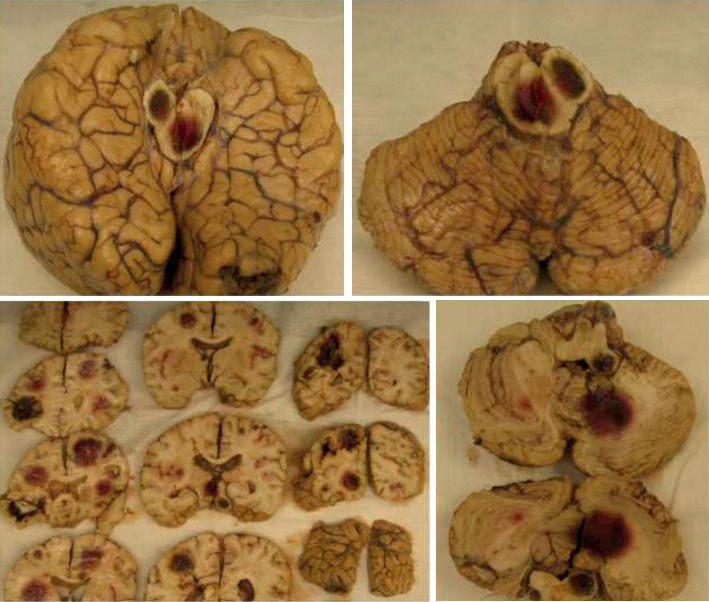
Autopsy brain. Multiple hemorrhagic necrotic lesions are present in the brain parenchyma including the cerebrum, cerebellum, and brainstem.

**Figure 3:**
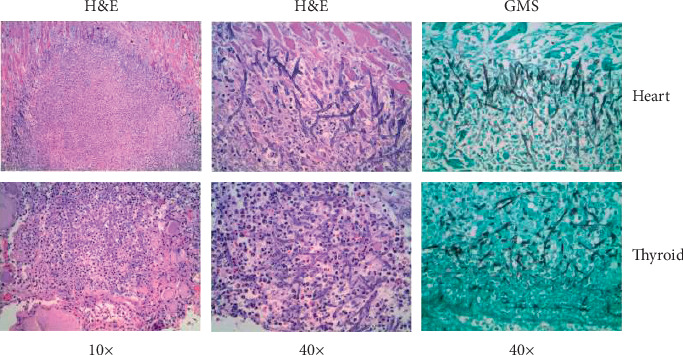
The heart and thyroid tissue display mycetomas. Fungal hyphae are spreading at the peripheral of the lesions. The center of the lesions shows prominent inflammation with neutrophilic infiltration and destruction of the cardiac myocytes (upper panel) and thyroid tissue (lower panel).

**Figure 4:**
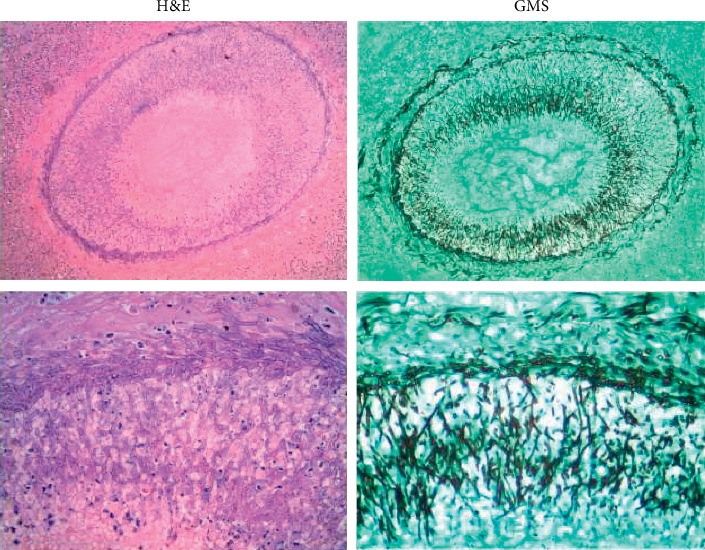
Active invading mycetoma is present in the occipital lobe, with 45-degree branching hyphae spreading horizontally at the peripheral and perpendicularly to the center.

At autopsy, pertinent gross findings included multiple small hemorrhagic skin papules on the chest, severe atherosclerotic coronary artery disease, multiple necrotic nodular lesions in the heart, thyroid, liver, and lung, and multiple hemorrhagic lesions in the brain (Figure 2). Microscopic examination of the lesions in the heart, thyroid, and to a lesser extent in the lung revealed the presence of 45-degree branching fungal hyphae and acute inflammation (Figure 3). In the hemorrhagic brain lesions, fungal balls (mycetomas) were identified with typical morphology of 45-degree branching hyphae spreading horizontally at the periphery and perpendicularly to the center (Figure 4). The necrotic nodular lesions of the liver were without identifiable fungus or other organisms. Skin sections revealed foci of the necrotic debris, and Gram stain and GMS stains were negative. Blood and lung cultures at autopsy were also negative. The cause of the patient's death was initially attributed to disseminated aspergillosis based on the morphologic characteristics. After the secondary review of the case, sequencing was performed and revealed the organism to be *S. boydii.*

## 5. Discussion

**Figure 5:**
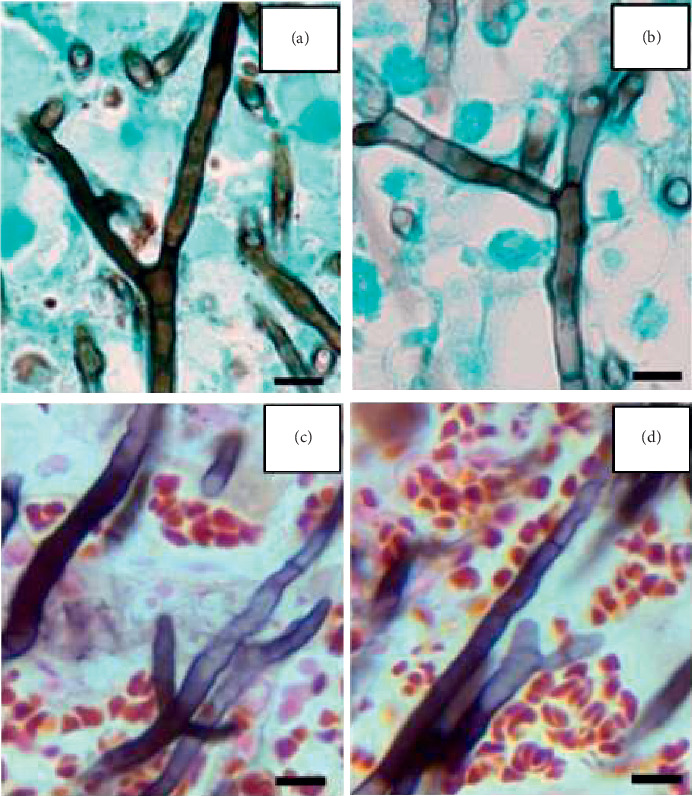
Comparison of fungal elements between two agents, both present with acute-degree branching hyphae. (a, b) Case report patient: *Scedosporium boydii* shows fork-shaped 45-degree branching hyphae. (c, d) Example *Aspergillus* patient: fungal hyphae demonstrating 45-degree alternating side branching in a biopsy specimen. Bar = 5 *μ*m.

*S. boydii (*previous naming-*P. boydii)* is a pathogenic species of the ascomycete genus *Scedosporium*. Members of the genus along with those of *Pseudallescheria* have been reclassified [6]. Other species include *S. auranticum, S. S. dehoogii, S. minutisporum*, and *S. desertorum.* The previously named pathogenic species *S. prolificans* has been renamed *Lomentospora prolificans* along with *L. inflatum*. These species are ubiquitous filamentous fungi present in soil, sewage, and polluted water. *S. boydii* can colonize airways of patients with existing disease such as poorly draining bronchi or paranasal sinuses, and “fungus ball” formation in preformed cavities is similar to that seen in *Aspergillus* [5, 7–9]. Infections caused by these organisms can be localized, extended to the surrounding tissues, or disseminated to distant organs by hematogenous spreading. The disseminated form of the disease is mostly seen among immunocompromised patients [8, 10]. Invasive infections of *S. boydii* in immunocompetent patients are usually caused by traumatic events such as following implantation and penetrating injuries [7, 8, 11]. Several types of infections have been described including pneumonia, arthritis, osteomyelitis, meningitis, brain abscesses, endophthalmitis, and disseminated systemic disease [7, 8, 10]. The diagnosis of Aspergillosis, one of the most frequently diagnosed fungal infections, is usually made by culture and/or evaluation of tissue sections or cytologic specimens. On histopathalogic or cytologic examination, the fungus demonstrates the presence of characteristic, septate fungal hyphae with 45-degree branching and an even diameter of 3–12 *µ*m (average 4.5 *µ*m) [12]. The major differential diagnosis of aspergillosis in surgical and cytologic specimens is mucormycosis which has thicker (5–20 *µ*m) and right-angle branching nonseptate hyphae [12]. In this report, the morphologic appearance of the *S. boydii* fungal hyphae in tissue sections closely resembles that of *Aspergillus* species (Figure 5) with 45-degree branching hyphae and an even diameter of 2.3–5.0 *µ*m (average 3.3 *µ*m). *Aspergillus* seems to have 45-degree alternating side branching, whereas *S. boydii* shows 45-degree fork-shaped branching hyphae, in this case (Figure 3). However, without culture or a secondary confirmatory test, misdiagnosis of aspergillosis can occur due to unawareness of infrequently occurring *S. boydii* as a potential pathogen and the close morphologic resemblance of the two organisms. Other uncommon molds can also mimic the appearance of *Aspergillus* species including *Fusarium, Trichoderma,* and *Paecilomyces* species [12]. Culture studies are critical to the diagnosis but when unavailable or inconclusive, sequencing of fungal rRNA genes for identification from surgical specimens is a very useful alternative. Scedosporiosis caused by *S. boydii* has a high mortality rate and is difficult to treat. Treatment of these infections is especially challenging because of the mold's resistance to many antifungal agents [13]. Although *S. boydii* resembles *Aspergillus* on pathologic examination, it is typically resistant to amphotericin B. Voriconazole has been shown as an effective agent to treat systemic *S. boydii* infection, while posaconazole shows less activity, and isavuconazole and itraconazole have minimal activity [4, 11–13]. Echinocandins have some degree of activity albeit at 5 to 10 times higher concentration than that used to treat *Aspergillus* [4]. In summary, this case highlights the importance of circumspection when diagnosing aspergillosis from anatomic pathology specimens based on morphologic characteristics, as therapeutic decisions may be suboptimal and result in added morbidity and mortality in these critically ill patients.

## Figures and Tables

**Table 1:** Abnormal laboratory test results.

Test	Result	Reference range
WBC	22.5	4–10 thou/mm^3^
pH	7.36	7.35–7.45
PCO_2_	32	35–45 mmHg
HCO_3_	18	23–27 mmol/L
Glucose	226	70–100 mg/dL
Sodium	128	136–146 meq/L
Potassium	7.3	3.4–5.2 meq/L
BUN	62	6–21 mg/dL
eGFR	12	>60 ml/min/1.73 sqm
Creatinine	6.1	0.64–1.27 mg/dL
AST	3522	10–39 IU/L
ALT	1986	4–42 IU/L
Myoglobin	235	0–90 ng/ml
Troponin	0.10	0.00–0.04 ng/ml
Lipase	92	6–32 units/L
APTT	37.4	25–35 sec
PT	19.3	12.3–14.8 sec
INR	1.6	0.91–1.14
D-dimer	>20	0.01–0.49 mcg/ml
FSP	>20	<5 mcg/ml
